# An exploratory study into the influence of laterality and location of hippocampal sclerosis on seizure prognosis and global cortical thinning

**DOI:** 10.1038/s41598-021-84281-y

**Published:** 2021-02-25

**Authors:** Alireza Mansouri, Jurgen Germann, Alexandre Boutet, Gavin J. B. Elias, Brij Karmur, Clemens Neudorfer, Aaron Loh, Mary Pat McAndrews, George M. Ibrahim, Andres M. Lozano, Taufik A. Valiante

**Affiliations:** 1 Department of Neurosurgery, Penn State Hershey Medical Center, Penn State University, 30 Hope Drive, Suite #1200, Hershey, PA 17033 USA; 2 University Health Network, Toronto, ON Canada; 3 Joint Department of Medical Imaging, University of Toronto, Toronto, ON Canada; 4 Krembil Research Institute, University Health Network, Toronto, ON Canada; 5 Division of Neurosurgery, The Hospital for Sick Children, Toronto, ON Canada; 6 Institute of Biomaterials and Biomedical Engineering, Department of Surgery, University of Toronto, Toronto, ON Canada; 7 Program in Neuroscience and Mental Health, Sickkids Research Institute, Toronto, ON Canada; 8 Division of Neurosurgery, Toronto Western Hospital, Toronto, ON Canada; 9 Krembil Research Institute, Toronto, ON Canada; 10 Institute of Biomaterials and Biomedical Engineering, University of Toronto, Toronto, ON Canada

**Keywords:** Epilepsy, Brain

## Abstract

In mesial temporal lobe epilepsy (mTLE), the correlation between disease duration, seizure laterality, and rostro-caudal location of hippocampal sclerosis has not been examined in the context of seizure severity and global cortical thinning. In this retrospective study, we analyzed structural 3 T MRI from 35 mTLE subjects. Regions of FLAIR hyperintensity (as an indicator of sclerosis)—based on 2D coronal FLAIR sequences—in the hippocampus were manually segmented, independently and in duplicate; degree of segmentation agreement was confirmed using the DICE index. Segmented lesions were used for separate analyses. First, the correlation of cortical thickness with disease duration and seizure focus laterality was explored using linear model regression. Then, the relationship between the rostro-caudal location of the FLAIR hyperintense signal and seizure severity, based on the Cleveland Clinic seizure freedom score (ccSFS), was explored using probabilistic voxel-wise mapping and functional connectivity analysis from normative data. The mean DICE Index was 0.71 (range 0.60–0.81). A significant correlation between duration of epilepsy and decreased mean whole brain cortical thickness was identified, regardless of seizure laterality **(**p < 0.05). The slope of cortical volume loss over time, however, was greater in subjects with right seizure focus. Based on probabilistic voxel-wise mapping, FLAIR hyperintensity in the posterior hippocampus was significantly associated with lower ccSFS scores (greater seizure severity). Finally, the right hippocampus was found to have greater brain-wide connectivity, compared to the left side, based on normative connectomic data. We have demonstrated a significant correlation between duration of epilepsy and right-sided seizure focus with global cortical thinning, potentially due to greater brain-wide connectivity. Sclerosis along the posterior hippocampus was associated with greater seizure severity, potentially serving as an important biomarker of seizure outcome after surgery.

## Introduction

Epilepsy is a brain network-wide phenomenon extending beyond the postulated seizure focus and mediated through functional connectivity hubs^1^. Prolonged epilepsy disease duration is known to be correlated with progressive functional decline and recent cross-sectional and prospective imaging-based studies suggest that aging-independent loss of cortical and subcortical volume, both globally and in specific functional hubs, may underlie this phenomenon^1,2^.

Among the various etiologies of epilepsy, mesial temporal lobe epilepsy (mTLE) has been subject to extensive investigations for imaging biomarker analyses^1–3^. The accumulating evidence has strengthened the notion that, like other forms of epilepsy, mTLE is a progressive disease associated with global atrophy and widespread cortical thinning^2,3^. The wide-ranging connectivity of the hippocampus to various functional networks within the brain suggests that the hippocampus may act as a central hub, mediating this progressive decline^4^. However, the relationship between this progression, and the laterality and rostro-caudal location of hippocampal sclerosis remain unknown.

In this cross-sectional analysis of people with mTLE secondary to hippocampal sclerosis, we sought to explore factors potentially associated with structural changes in the epileptic brain and seizure severity, as parsing out these relationships may have implications on clinical decision making (e.g. if and when to perform surgical intervention). Our primary objective was to assess the impact of seizure duration, along with laterality of hippocampal sclerosis, on changes in global cortical thickness. Secondarily, we sought to determine whether laterality and rostro-caudal location of hippocampal sclerosis correlated with seizure severity. Finally, based on the hypothesis of epilepsy as a brain-wide phenomenon, differences in global connectivity between the left and right hippocampi were explored.

## Methods

This retrospective, single-center, cross-sectional study focused on individuals with (1) a preoperative clinical history consistent with mTLE, (2) imaging presentation of clear area(s) of FLAIR signal hyperintensity in the hippocampus consistent with the side of seizure focus, and (3) pathological confirmation of hippocampal sclerosis following surgical resection. FLAIR signal hyperintensity has been commonly used as a surrogate for hippocampal sclerosis in imaging investigations for epilepsy, although it is known that that the seizure onset zone spans beyond the visible sclerotic region(s)^5–7^. This was a retrospective study conducted at the University Health Network following institutional research ethics board approval (study ID UHN13-6399). All research was performed in accordance with relevant guidelines/regulations. As approved by the board, informed consent was waived due to the retrospective nature of the study.

### Segmentation of hippocampal sclerosis

**Figure 1:**
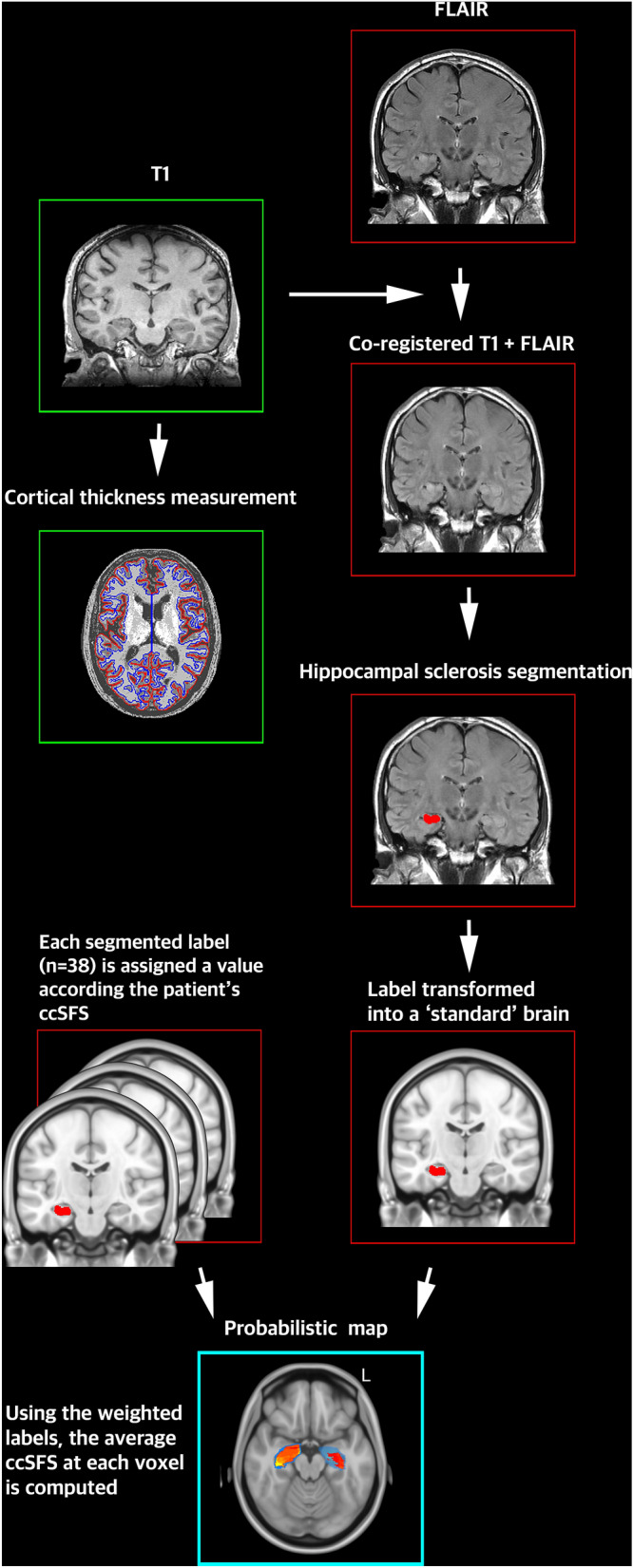
Summary of methods. For each patient, a T1 sequence and FLAIR sequence were co-registered. Using the co-registered T1 + FLAIR, areas of hippocampal hyperintensity—corresponding to hippocampal sclerosis—were segmented. The resulting label was transformed into 'standard' space, in order to facilitate group level analysis. This process was completed for all 38 patients, resulting in 38 labels. Each label was assigned a value according to each patient's ccSFS. Using all 38 weighted labels, the average ccSFS at each voxel was computed in the 'standard brain', producing the probabilistic map. Cortical thickness for each patient was computed using their T1 images, using the program CIVET. *ccSFS* Cleveland Clinic seizure freedom score, *FLAIR* fluid attenuated inversion recovery, *T1* T1 weighted.

The preoperative MRI conducted on the day of surgery for intraoperative navigation purposes was used for lesion segmentation in all cases. Coronal FLAIR sequences were used to segment regions of hippocampal sclerosis. In order to permit more accurate delineation and subsequent normalization, clinically-relevant coronal FLAIR images (1 × 1 × 4 mm TE = 100 ms, TR = 9000 ms) were first registered (12 degrees of freedom) and up-sampled (using b-spline interpolation) to a corresponding preoperative T1-weighted 3D-SPGR acquisition (isotropic 1 × 1 × 1 mm voxel, TE = 3 ms, TR = 8 ms) (https://github.com/BIC-MNI/minc-toolkit-v2) (Supplementary Table 1). A graphical summary of this approach has been outlined in Fig. 1. From posterior to anterior, hyperintense segments of the hippocampus were segmented, slice by slice, until no further hyperintense signal could be seen. Segmentation was done on the registered and up-sampled coronal FLAIR first by an attending neurosurgeon with expertise in epilepsy surgery (AM) and corroborated by a second independent rater (AL) to validate the segmentation accuracy and validity. The degree of segmentation agreement was assessed based on the Dice Index, a metric that evaluates overlap between binarized volumes according to the following formula:

$$ DSC = \frac{{2\left| {X \cap Y} \right|}}{\left| X \right| + \left| Y \right|} $$

In this formula, |X| and |Y| are the cardinalities of the two sets (i.e. the number of elements in each set). The Sørensen index equals twice the number of elements common to both sets divided by the sum of the number of elements in each set. Using this approach, the existing literature reports average values between 0.6 and 0.8 when manually segmenting FLAIR lesions^9,10^.

Further, the head body and tail sections of the hippocampus were identified (anterior to posterior) based on the presence of uncal apex (head), presence of thalamus in the coronal slice (body) and region posterior of the thalamus (tail)^11^. Presence/absence of sclerosis to head, body or tail of the hippocampus was recorded in a binary code (yes/no) for further analysis.

### Correlation of whole brain cortical thickness with disease duration and seizure focus laterality

Cortical thickness measurements were obtained using CIVET (Montreal Neurological Institute). Briefly, T1-weighted patient images were preprocessed using the minc-bPIPE (https://github.com/CobraLab/minc-bpipe-library) library and nonlinearly registered to MNI152 space^12^. Tissue classification was then performed using patient-specific registration priors^13^. The white matter and pial surfaces were extracted and registered to the MNI152 surface template. Cortical thickness was then computed at each vertex based on the distance between grey and white matter surfaces (Fig. 1)^14^. Whole brain mean cortical thickness was computed for each subject and the correlation with seizure focus laterality potential predictors of whole brain cortical thickness explored using linear models.

### Probabilistic map for prognosis of postoperative seizure freedom

In order to investigate whether volume, laterality, and location of hippocampal sclerosis—based on FLAIR signal hyperintensity—may correlate with seizure severity, we constructed probabilistic maps based on the Cleveland Clinic seizure freedom score (ccSFS) for participants undergoing surgery. The ccSFS is a clinical prediction tool used to predict the likelihood of postoperative seizure freedom. In this 5-point scale (0 to 4), the categorical variables of preoperative seizure frequency (greater or less than 20 seizures per month), history of generalized tonic–clonic seizures, presence of abnormality on brain MRI, and duration of epilepsy (greater or less than 5 years) are assessed preoperatively. A lower score is indicative of a lower likelihood of postoperative seizure freedom and thus an indicator of disease severity^15^.

To enable group-level analysis, all T1-weighted images were normalized to standard space (ICBM 2009b NLIN asymmetric) in a two-step process comprising 9 degrees of freedom linear registration (FLIRT) followed by nonlinear registration (FNIRT) with the previously segmented masks used as input-weighing volumes^16^. For each step, the previously segmented FLAIR hyperintensity masks were used as input-weighting volumes to avoid registration distortion. This transformation was then applied to the FLAIR hyperintensity masks. Segmented lesions were weighted by their respective ccSFS scores (correlate of disease severity) and normalized by size to penalize larger and less focal lesions. Subsequently, the “risk of ongoing seizures” (based on the mean ccSFS) at each voxel was computed by averaging the normalized weighted values of all overlapping lesion maps (i.e., average map, Fig. 1). The resultant average map was then masked by a frequency map that captured the total number of lesion masks in contact with each voxel, and thresholded at 10% to exclude outlier voxels. Finally, a statistical map was calculated using Wilcoxon signed-rank tests to determine, at each voxel, the degree of confidence in the association between FLAIR signal hyperintensity and ccSFS scores (null hypothesis: there is no association with ccSFS score at a given voxel). Each average map was masked by the corresponding statistical map, thresholded at *p* < 0.05^17^. In other words, each map defines the probability of having higher or lower ccSFS score based on the segmented FLAIR labels in our cohort.

### Exploring differences in brain-wide connectivity based on seizure focus laterality

Greater seizure severity may be related to greater brain-wide connectivity of the seizure focus. Because the patient’s included in our study did not have native functional imaging acquisitions, patterns of functional connectivity associated with seizure focus laterality were explored using an established functional connectivity mapping method that has been leveraged in numerous previous studies^19–23^. This method uses a large-scale, high-quality normative resting-state fMRI (rsfMRI) dataset constructed from 1000 healthy subjects (http://neuroinformatics.harvard.edu/gsp). Detailed information about the preprocessing and aggregation of this dataset have been previously described^18–24^. Briefly, each healthy subject in the normative dataset was scanned once or twice (1.7 times per subject on average) with a 6.2 min-long echo-planar imaging sequence (124 time points; 3 × 3 × 3 voxel size, TR 3000 ms, TE 30 ms, flip angle 85°) in order to acquire rsfMRI data. While the functional connectivity of brain regions may differ in the diseased state—potentially limiting the direct translatability of our findings^25^—normative connectivity datasets are derived from a large number of subjects using specialized MRI hardware and acquisition parameters, resulting in a highly reliable and reproducible connectivity pattern, superior to single-subject native rsfMRI. Furthermore, prior work has demonstrated that normative data can be reliably used for localization of neurological disorders, including epilepsy^25,26^.

For connectomic analysis, individual FLAIR hyperintensity masks were treated as seeds. Then, a connectivity r-map describing the correlation between the seed and every voxel in the brain—on the basis of the averaged low-frequency blood-oxygen-level-dependent (BOLD) signal fluctuations sampled across the subjects in the normative dataset—was obtained (in-house MATLAB script, The MathWorks, Inc., Version R2017b. Natick, MA, USA). To investigate differences of local connectivity strength between left- and right-sided seizure foci, a whole-brain voxel-wise logistic regression was performed. The resulting t-stat map was corrected for multiple comparisons using False Discovery Rate at the voxel level (thus at p_FDRcor_ < 0.01 at least 99 out of every 100 voxels are true positives)^27^.

### Exploring relationship between cortical thickness decline and seizure focus

To investigate possible determinants of cortical thinning, linear models of the relationship between individual mean cortical thickness and age, disease duration, seizure laterality, volume and location (head/body/tail) of hippocampal sclerosis were calculated.

### Statistical analysis

Statistical analyses were performed using Python (Python 3.6.5, https://www.python.org/download/releases/365/), R (R 3.4.4, https://www.r-project.org; rstudio 1.1.463, https://www.rstudio.com/), and RMINC (https://github.com/Mouse-Imaging-Centre/RMINC).

## Results

### Subjects

Thirty-five individuals with mTLE and distinct FLAIR signal hyperintensities that correlated with the side of seizure focus (20 left and 15 right) were included. At 1-year postoperative follow-up, 29/35 (83%) subjects had Engel 1 seizure status, 2/35 (5.7%) had Engel 2, 2/35 (5.7%) were Engel 4, and in 2/35 seizure status was unknown (Table 1). There was no significant correlation between the 1-year Engel 1 status and the ccSFS. The mean DICE index for the degree of agreement between segmented regions by the two authors was 0.71 (range 0.70–0.81, standard deviation 0.06), indicative of good agreement. There was no statistically-significant difference between the mean (± SD) left and right side segmented volumes (0.9 ± 0.4 cc versus 1.1 ± 0.6 cc, p = 0.3) and no correlation was found between FLAIR volume and individual 1-year Engel 1 seizure status or ccSFS score.

**Table 1:** Baseline and postoperative parameters of subjects analyzed in study.

Variable	Left MTS (N = 20)	Right MTS (N = 15)
**Female, N (%)**	9 (45%)	7 (46%)
**Median age at onset (range)**	4.5 (0.5–38)	14 (2–49)
**Median age at surgery (range)**	37.5 (21–59)	38 (25–65)
**Median years to surgery (range)**	13 (5–31)	16 (2–46)
**Cleveland clinic seizure freedom scores**		
0	1	0
1	9	3
2	8	9
3	2	2
4	0	1
**Engel-1 status at 1-year, N (%)**	16 (80%)	13 (87%)

### Correlation of whole brain cortical thickness with disease duration and seizure focus laterality

**Figure 2:**
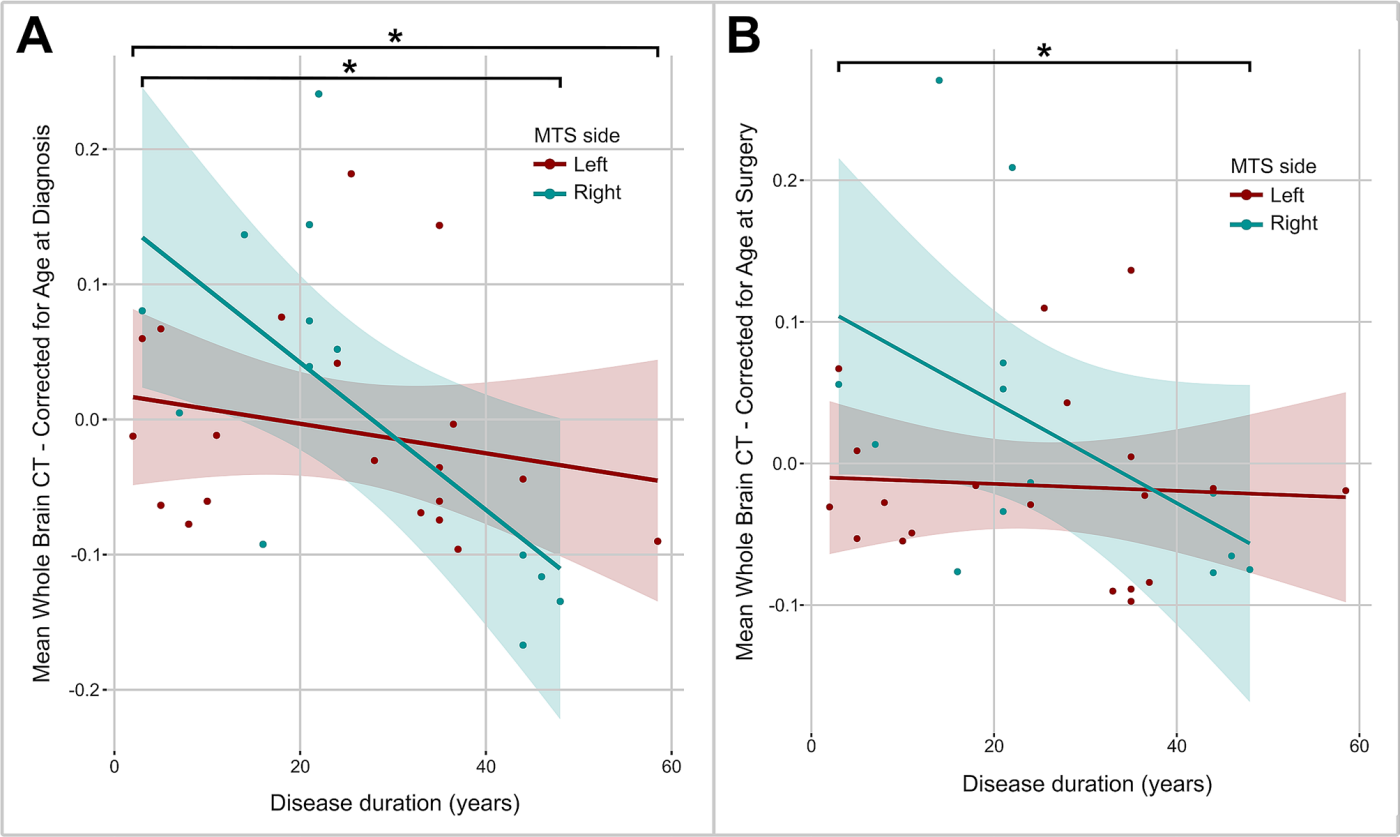
Whole brain cortical thickness decrease is related to disease duration and seizure focus laterality. (**A**) The relationship between disease duration and whole brain cortical thickness after correcting for age at diagnosis. (**B**) Relationship between disease duration and whole brain cortical thickness after correcting for age at surgery.

Using age at diagnosis as a covariate, a significant correlation between duration of epilepsy and decrease in cortical thickness for both left and right seizure foci was identified **(**Fig. 2A, p = 0.01 for both), with right seizure focus subjects featuring accelerated decline (greater slope). This analysis was done to differentiate normal aging from disease processes leading to cortical thinning over time. Given the possible covariance of age with disease duration—older individuals potentially having had a longer history of epilepsy—we also assessed this correlation with age at surgery as a covariate instead. Through this approach, disease duration remained significantly associated with decreased cortical thickness only in the right seizure focus group (Fig. 2B, p = 0.05). Individual FLAIR volume did not have an impact on cortical thickness changes. There was a trend toward an accelerated rate of cortical thinning in individuals with posterior hippocampal sclerosis if the seizure focus was located on the right **(**p = 0.03; interaction of epilepsy duration and tail sclerosis: p = 0.08).

### Probabilistic map for prognosis of postoperative seizure freedom

**Figure 3:**
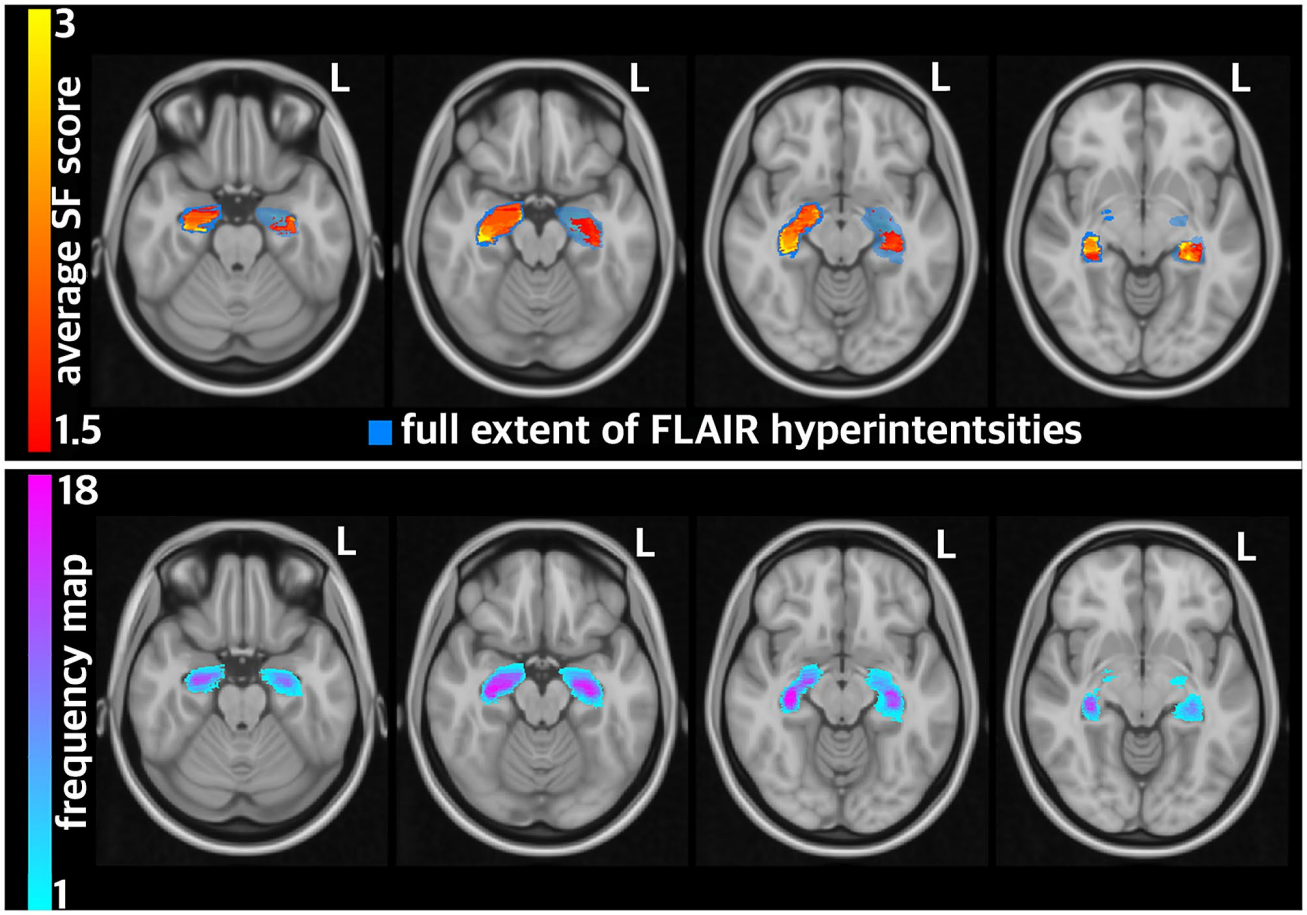
Probabilistic mapping of seizure risk using FLAIR signal hyperintensity regions. Areas of hyperintense FLAIR signal were segmented in each patient, transformed into a common brain (MNI152), and then weighted by their corresponding ccSFS score. The average risk of seizure based on the ccSFS score and FLAIR signal location was then computed at each voxel (warm colors). This probabilistic map of seizure risk (warm colors) is overlaid on maps showing the general distribution of the FLAIR signal across patients (i.e., summation of frequency maps, cool colors). For both sides, the highest risk of seizures was seen in the posterior hippocampus.

To investigate whether the pattern of global cortical thinning had ramifications on- or otherwise related to—seizure severity, we examined the correlation between the ccSFS as a surrogate of preoperative seizure severity and location of FLAIR hyperintensity. In doing so, we found that FLAIR hyperintensities located in the posterior hippocampus were significantly associated with lower ccSFS scores, indicating that in parallel with global cortical thinning, FLAIR hyperintense lesion (sclerosis) overlap with this region portends greater disease severity as well (Fig. 3).

### Exploring differences in brain-wide connectivity based on seizure focus laterality

**Figure 4:**
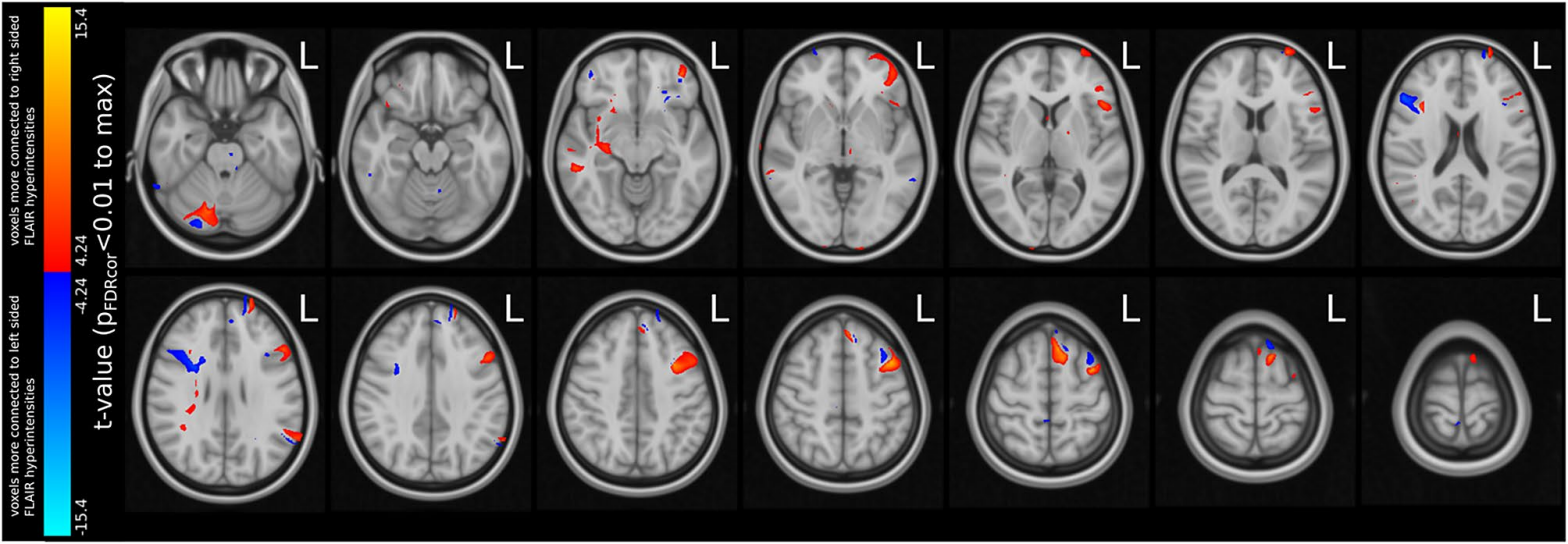
Comparing the ‘connectedness’ (normative connectome) of right vs left seizure focus FLAIR volumes. All colored areas—overlaid on standard brain template (MNI152)—are significantly (FDR < 0.01, i.e. 99 out of every 100 voxels is a true positive) more connected to regions of either right-sided hippocampal FLAIR hyperintensity (warm colors) or left-sided hippocampal FLAIR hyperintensity (cold colors).

Given the hypothesis that epilepsy is a brain network-wide phenomenon and that increased brain-wide connectivity may portend a lower likelihood of postoperative seizure freedom, we performed a direct comparison between the brain network-wide connectivity of right and left-sided FLAIR hyperintense segments. Through this post-hoc analysis, we found that the regions encompassed by right-sided seizure foci exhibited greater overall brain-wide connectivity in normative data than those associated with left-sided foci (p_FDRcor_ < 0.01) (Fig. 4).

## Discussion

Our study reaffirms epilepsy as a brain network-wide phenomenon that leads to progressive global cortical thinning. In our cohort, this progressive decline appeared to be driven to a greater extent by right-sided pathology, with additional influence from FLAIR signal abnormality within the posterior hippocampus. This pattern correlated with greater cortical connectivity of this region as well as lower preoperative ccSFS scores, suggesting a possible interplay between these factors.

Accelerated cortical thinning, independent of normal aging effects, has been demonstrated in individuals with any form of epilepsy and right-sided mTLE individuals as subgroups; however, the impact of laterality and rostro-caudal location of hippocampal sclerosis has not been assessed in prior work^28^. Our results suggest that right-sided sclerosis accelerates cortical thinning, and that this could be correlated with underlying differences in brain network-wide connectivity between the left and right hippocampus. Furthermore, given the emerging understanding of the long-axis anatomical and functional differences within the hippocampus, it is likely that the exact location of hippocampal sclerosis may be prognostic^8^. Several prior investigations have reported on progressive cortical/ sub-cortical (both general and focal) thinning in epilepsy with some linking this correlation with the laterality of hippocampal sclerosis. While these investigations have offered great insight our methods were based on an unbiased approach in order to examine the “risk” of ongoing postoperative seizures (based on the ccSFS) at each FLAIR hyperintense voxel^2,28,29^. Furthermore, this study is the first to report on the potentially variable impact of sclerosis along the rostro-caudal axis of the hippocampus.

Using the ccSFS tool as a surrogate for seizure severity, we found a significant correlation between posterior right hippocampal sclerosis and the likelihood of poor postoperative seizure outcome. Furthermore, the posterior hippocampus on the right side was found to have a greater brain-wide connectivity, compared to the left side, based on normative connectivity data. Although it is now understood that greater extent of hippocampal resection may translate to better seizure outcomes, the optimal hippocampal resection volume currently remains unknown^30^. The presence of FLAIR signal abnormality may thus serve as a guide for the posterior limit of hippocampal resection in these cases.

### Limitations

Our findings should be interpreted with caution due to the retrospective nature of the study and the small sample size. A high proportion of our subjects achieved Engel 1 seizure status at 1-year, which limited our ability to conduct a meaningful statistical and neuroimaging analysis based on actual seizure outcomes and to reliably compare with the ccSFS. The ccSFS tool was derived based on a large subset of surgical patients with several years of postoperative follow-up. Indeed, while preoperative ccSFS scores have shown a strong correlation with actual postoperative seizure freedom at 10 year follow-up^15^, the correlation of ccSFS with seizure freedom at shorter postoperative timepoints (e.g. 1 year) is less well established. Even though we found no significant correlation between ccSFS and Engel 1 seizure status at 1-year, it is plausible that ccSFS and Engel status would have shown more agreement if Engel status at a later time point was performed. Longer-term follow up of a larger cohort is needed for a more definite assessment of the true correlation between postoperative seizure outcomes and location of hippocampal sclerosis. In addition, our connectomic analysis was based on an extrapolation of FLAIR hyperintense signal within a diseased hippocampus to an atlas of healthy volunteers. However, the objective of our analysis was to provide a possible mechanism for differences in cortical volume based on laterality and location of sclerosis in the hippocampus. Even so, future studies should attempt to leverage patient or disease specific functional imaging data to perform similar connectomic analysis.

Furthermore, we opted to focus exclusively on subjects with clear FLAIR signal abnormality, which despite demonstrating a good correlation with pathological gliosis, is not an absolute marker. In addition, other factors including the volume and functional integrity of other temporal lobe structures, such as the parahippocampal gyrus, and other medical comorbidities or impact of anticonvulsant medications were not taken into consideration. There is a clear need for prospective longitudinal studies to assess for dynamic changes in cortical volume with time and to examine the potential validity of laterality/location of hippocampal sclerosis as an imaging biomarker of surgical success.

## Conclusion

Our results suggest that both laterality of seizure focus and location along the rostro-caudal hippocampal axis are important factors affecting the detrimental whole brain sequelae of epilepsy. Given the accelerated generalized whole brain cortical thickness loss seen in patients with right-sided MTS, particularly those with FLAIR hyperintense signal in the posterior hippocampus, these patients should be considered for earlier referral for surgical assessment and intervention. Furthermore, in addition to the laterality of hippocampal FLAIR hyperintensity, the specific location of the FLAIR hyperintense signal along the rostro-caudal axis of the hippocampus should also be taken into consideration when assessing urgency of surgical intervention, as this could be a valuable imaging biomarker in epilepsy.

## Supplementary Information

Supplementary information.

## Data Availability

Pertaining to the current study, anonymized data will be shared by request from any qualified investigator.
