# Supplementary material for: An exploratory study into the influence of laterality and location of hippocampal sclerosis on seizure prognosis and global cortical thinning
Source: Sci Rep. 2021 Feb 25;11:4686. doi: 10.1038/s41598-021-84281-y (PMC7907189; doi:10.1038/s41598-021-84281-y)
Supplement: Supplementary file 1 — Supplementary information. [file 41598_2021_84281_MOESM1_ESM.pdf]

An exploratory study into the influence of laterality and location of hippocampal sclerosis on seizure prognosis and global cortical thinning

Alireza Mansouri*[1], Jurgen Germann*[2], Alexandre Boutet[2,3], Gavin J.B. Elias[2], Brij Karmur[2], Clemens Neudorfer[2], Aaron Loh[2], Mary Pat McAndrews[4], George M. Ibrahim[5,6,7], Andres M. Lozano[8,9], Taufik A. Valiante[8,9,10]

Tables

Supplementary Table 1. MRI Acquisition parameters.

Sequence	Tesla	TR (ms)	TE (ms)	TI (ms)	ETL	FA (degrees)	slice thickness (mm)	gap value (mm)	matrix
T1w 3D SPGR	3	8	3	450	1	12	1	0	256 x 256
2D coronal FLAIR	3	9000	100	2250	1	90	4	1	352 x 224

ETL = echo train length; FA = flip angle; FLAIR = fluid attenuated inversion recovery; SPGR = spoiled gradient-recalled; TE = echo time; TI = inversion time; TR = repetition time
